# Clinical Audit of Use of Antipsychotics in Generic CAMHS Redcar in the Context of Baseline Investigations and Monitoring

**DOI:** 10.1192/bjo.2025.10653

**Published:** 2025-06-20

**Authors:** Fatma Sena Ozbal, Lisa Egglestone

**Affiliations:** 1 Tees, Esk and Wear Valleys NHS Foundation Trust, Middlesbrough, United Kingdom; 2 Tees, Esk and Wear Valleys NHS Foundation Trust, Redcar, United Kingdom

## Abstract

**Aims:** The data on safety of antipsychotics in children and young people (CYP) remains insufficient, mainly because this age group usually is not included in trials. It is also partially because there are relatively low numbers of CYP on antipsychotics. Use of antipsychotics come with risks of short- and long-term side effects, the cumulative side effects are particularly important in CYPs. Baseline investigation and regular monitoring are crucial to identify any underlying or emergent health conditions to ensure CYP’s safety.

We aimed to evaluate our clinic’s compliance with the standards to ensure patients receive the necessary care.

**Methods:** The standards for this audit are based on “NICE – Guidance on Baseline Investigations and Monitoring for Children and Young People Prescribed Antipsychotic Medication” and “TEWV (2024) Psychotropic Monitoring Guide (Pharm-0082-V3.2) – Antipsychotics (Page 3 and 4)”.

Eligibility criteria included CYP on the Redcar CAMHS caseload between the dates 2/9/24–30/9/24, prescribed an antipsychotic with or without other psychotropics. All cases meeting eligibility criteria were included, with eligible samples identified through communicating with team members including team manager and prescribers. Seven CYP aged between 10–17 were identified eligible for the audit.

An audit tool was used in the data collection.

CITO (local electronic system) case notes and clinic letters are used as source of data. Search of key words such as “antipsychotic”, “aripiprazole”, “risperidone” also facilitated.

**Results:** The overall compliance rate was below 50% for this audit.

Areas of good practice: 80% compliance in the assessment of side effects at 3-month interval and 100% compliance in consideration of ECG, and completion of ECG if required, at 12-month interval.

Summary of issues identified: Poor compliance (below 80%) in recording weight, height, growth chart (or centile), waist circumference, blood pressure (BP) and heart rate (HR) checks, blood tests at the corresponding intervals, and pre-treatment ECG consideration or undertaking investigation.

**Conclusion:** In CAMHS, transitions between mental health services and patients being open to more than one team situations are common, often leading to confusion about responsibilities. Poor handover processes between teams can contribute to low compliance. Therefore, teams to improve their handover practices.

Due to small number of patients on antipsychotics in CAMHS, we do not have physical health check clinic within the team. Therefore, some of the physical health checks are requested from the GP colleagues (particularly blood tests and ECG). However, communication is not always immediate, making it difficult to track the results or document them. This can lead to missed or overlooked data. The communication and documentation to be more elaborate.

There is also no clear consensus on where to record monitoring data. For instance, while the Physical Health Tab on CITO is consistently used by some teams, this is not the case for my team. Additionally, some monitoring data is recorded in progress notes but not in the clinic letters. This issue to be discussed within the CAMHS team for clarification.

